# Maximized directed information transfer in critical neuronal networks

**DOI:** 10.1186/1471-2202-12-S1-P18

**Published:** 2011-07-18

**Authors:** Mikail Rubinov, Joseph Lizier, Mikhail Prokopenko, Michael Breakspear

**Affiliations:** 1 School of Psychiatry, University of New South Wales, Sydney, NSW 2031, Australia; 2 Mental Health Research Division, Queensland Institute of Medical Research, Brisbane, QLD 4029, Australia; 3 CSIRO Information and Communication Technologies Centre, Sydney, NSW 1710, Australia; 4 Max Planck Institute for Mathematics in the Sciences, Leipzig 04103, Germany

## 

Critical dynamics in complex systems emerge at the transition from random to ordered dynamics and are characterized by power-law distributions of spatial and temporal properties of system events. The occurrence of critical dynamics in neuronal networks is increasingly supported by multielectrode array recordings of spontaneous activity in organotypic cortical slice cultures [1]. System events in these neuronal networks are typically defined as activations of neuronal ensembles, or “neuronal avalanches”. Interestingly, studies associate critical neuronal network avalanche dynamics with optimized information transfer [1,2]. However, studies have not previously examined the directed nature of information transfer in these networks.

Here, we present three novel transfer-entropy [3] based measures of directed information transfer between neuronal avalanches. Our measures compute the amount of predictive information present in avalanches properties (avalanche size, avalanche duration and inter-avalanche period) of the source region about avalanche properties of the destination region and are suitable for detecting information transfer at multiple spatial scales, from individual neurons to neuronal ensembles. We apply these measures to compute directed information transfer in large, sparse, modular networks of leaky integrate-and-fire neurons with spike timing-dependent synaptic plasticity and axonal conduction delays. We characterize dynamics in our networks by distributions of neuronal avalanches and assess these distributions for power-law scaling. We compute directed information transfer between two halves of each network, and we normalize this transfer by the null-model transfer, generated by randomly rotating avalanche times for each avalanche vector, and so destroying any present relationships between groups of vectors.

**Figure 1:**
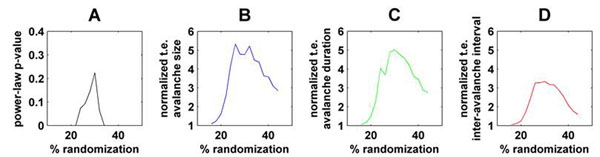
Directed information transfer at criticality. Fig 1A shows the presence of critical dynamics as a function of network randomization; p-values that exceed 0.5 imply power-law distributions of avalanches. Fig 1B-D show our normalized directed information transfer (transfer entropy, t.e.) between two halves of the network, based on avalanche size (blue), avalanche duration (green) and inter-avalanche interval (red) system properties.

Dynamics in our networks change from subcritical to critical to supercritical as the modular topology of the networks is progressively randomized (Fig. 1a). All three measures peak at criticality in all examined networks (Fig 1b-d) and hence show that directed neuronal information transfer is maximized at criticality in our model. These findings pave the way for the application of our measures to empirical multielectrode recording data.
